# Supplementary material for: Assessment of Grouped Weighted Quantile Sum Regression for Modeling Chemical Mixtures and Cancer Risk
Source: Int J Environ Res Public Health. 2021 Jan 9;18(2):504. doi: 10.3390/ijerph18020504 (PMC7827322; doi:10.3390/ijerph18020504)

International Journal of
Environmental Research and Public Health

MDPI

Assessment of Grouped Weighted Quantile Sum Regression for Modeling Chemical Mixtures and Cancer Risk

Supplemental Material

Table S1. List of Chemicals and Their Group used in the CCLS Analysis.

Chemical	Chemical Group
pcb_118	PCB
pcb_138	PCB
pcb_153	PCB
pcb_180	PCB
p.p.dde	Insecticide
p.p.ddt	Insecticide
cyfluthrin_i	Insecticide
cyfluthrin_ii	Insecticide
cyfluthrin_iii	Insecticide
cyfluthrin_iv	Insecticide
carbaryl	Insecticide
propoxur	Insecticide
pentachlorophenol	Insecticide
chlordane1	Insecticide
chlordane2	Insecticide
chlorpyrifos	Insecticide
diazinon	Insecticide
phosmet	Insecticide
cis.permethrin	Insecticide
methoxychlor	Insecticide
cypermethrin_i	Insecticide
cypermethrin_ii	Insecticide
cypermethrin_iii	Insecticide
cypermethrin_iv	Insecticide
trans.permethrin	Insecticide
piperonyl_butoxide	Insecticide
o.phenylphenol	Herbicide
trifluralin	Herbicide
simazine	Herbicide
mcpp	Herbicide
dicamba	Herbicide
dacthal	Herbicide
h2.4.d	Herbicide
as	Metals
cr	Metals
cu	Metals
pb	Metals
sn	Metals
w	Metals

zn	Metals
indeno.123cd.pyrene	PAH
dibenz.ah.anthracene	PAH
dibenzo.ae.pyrene	PAH
coronene	PAH
benzo.a.anthracene	PAH
benzo.a.pyrene	PAH
benzo.b.fluoranthene	PAH
nicotine	Tobacco
cotinine	Tobacco

Table S2. True and Estimated Odds Ratios for the Four Models for Scenario C.

Parameter	GWQS	WQS	Grouped Lasso	Lasso
Weak Correlation				
exp(β_1)= 1.00	0.9985		0.9923	
exp(β_2)= 1.00	0.9921	0.9887	0.9909	0.99
exp(β_3)= 1.00	1.0144		1.0071	
exp(β_1)= 0.67	0.7125		0.7234	
exp(β_2)= 1.50	1.5164	1.8949	1.4600	1.50
exp(β_3)= 1.50	1.4743		1.4330	
exp(β_1)= 0.50	0.5286		0.5260	
exp(β_2)= 2.00	2.0495	3.0105	1.9632	1.96
exp(β_3)= 2.00	1.9776		1.9480	
exp(β_1)= 0.40	0.4173		0.4168	
exp(β_2)= 2.50	2.5790	4.2653	2.4676	2.48
exp(β_3)= 2.50	2.5319		2.4847	
exp(β_1)= 0.33	0.3496		0.3455	
exp(β_2)= 3.00	3.0820	5.3863	2.9880	3.00
exp(β_3)= 3.00	3.3066		3.0339	
Moderate Correlation				
exp(β_1)= 1.00	1.0047		0.9949	
exp(β_2)= 1.00	0.9930	0.9966	0.9924	0.99
exp(β_3)= 1.00	1.0112		1.0036	
exp(β_1)= 0.67	0.6910		0.7082	
exp(β_2)= 1.50	1.4953	1.6864	1.4635	1.49
exp(β_3)= 1.50	1.4839		1.4462	
exp(β_1)= 0.50	0.5220		0.5178	
exp(β_2)= 2.00	1.9817	2.3550	1.9706	1.97
exp(β_3)= 2.00	1.9599		1.9574	
exp(β_1)= 0.40	0.4223		0.4121	
exp(β_2)= 2.50	2.4791	2.9847	2.4897	2.46
exp(β_3)= 2.50	2.4450		2.4660	
exp(β_1)= 0.33	0.3524		0.3393	
exp(β_2)= 3.00	2.9867	3.5393	3.0164	2.97
exp(β_3)= 3.00	2.9301		3.0038	
Strong Correlation				
exp(β_1)= 1.00	1.0030	0.9887	0.9948	0.99
exp(β_2)= 1.00	0.9941		0.9948	

$\exp(\beta_3)$= 1.00	1.0123		1.0047	
$\exp(\beta_1)$= 0.67	0.6902		0.6926	
$\exp(\beta_2)$= 1.50	1.4935	2.1012	1.4714	1.49
$\exp(\beta_3)$= 1.50	1.4974		1.4805	
$\exp(\beta_1)$= 0.50	0.5187		0.5070	
$\exp(\beta_2)$= 2.00	1.9857	3.3858	1.9863	1.97
$\exp(\beta_3)$= 2.00	1.9879		1.9942	
$\exp(\beta_1)$= 0.40	0.4192		0.4023	
$\exp(\beta_2)$= 2.50	2.4360	4.4484	2.4993	2.47
$\exp(\beta_3)$= 2.50	2.5052		2.5221	
$\exp(\beta_1)$= 0.33	0.3513		0.3328	
$\exp(\beta_2)$= 3.00	2.9141	5.3476	3.0304	2.97
$\exp(\beta_3)$= 3.00	2.9710		3.0404	

Table S3. Power and Type I Error for the Four Models for Scenario C.

Parameter	GWQS	WQS	Grouped Lasso	Lasso
Weak Correlation				
$\exp(\beta_1)$= 1.00	0.09		(0.02, 0.00)	
$\exp(\beta_2)$= 1.00	0.09	0.07	(0.07, 0.00)	(0.34, 0.00)
$\exp(\beta_3)$= 1.00	0.05		(0.07, 0.00)	
$\exp(\beta_1)$= 0.67	0.81		(0.97, 0.33)	
$\exp(\beta_2)$= 1.50	0.97	0.97	(1.00, 0.36)	(1.00, 0.52)
$\exp(\beta_3)$= 1.50	0.85		(0.91, 0.28)	
$\exp(\beta_1)$= 0.50	1.00		(1.00, 0.97)	
$\exp(\beta_2)$= 2.00	1.00	1.00	(1.00, 0.99)	(1.00, 1.00)
$\exp(\beta_3)$= 2.00	1.00		(1.00, 0.96)	
$\exp(\beta_1)$= 0.40	1.00		(1.00, 1.00)	
$\exp(\beta_2)$= 2.50	1.00	1.00	(1.00, 1.00)	(1.00, 1.00)
$\exp(\beta_3)$= 2.50	1.00		(1.00, 1.00)	
$\exp(\beta_1)$= 0.33	1.00		(1.00, 1.00)	
$\exp(\beta_2)$= 3.00	1.00	1.00	(1.00, 1.00)	(1.00, 1.00)
$\exp(\beta_3)$= 3.00	1.00		(1.00, 1.00)	
Moderate Correlation				
$\exp(\beta_1)$= 1.00	0.09		(0.05, 0.02)	
$\exp(\beta_2)$= 1.00	0.05	0.07	(0.04, 0.00)	(0.27, 0.00)
$\exp(\beta_3)$= 1.00	0.06		(0.02, 0.01)	
$\exp(\beta_1)$= 0.67	0.93		(0.88, 0.61)	
$\exp(\beta_2)$= 1.50	0.98	1.00	(0.91, 0.56)	(0.99, 0.71)
$\exp(\beta_3)$= 1.50	0.94		(0.84, 0.61)	
$\exp(\beta_1)$= 0.50	1.00		(0.97, 0.99)	
$\exp(\beta_2)$= 2.00	1.00	1.00	(1.00, 0.98)	(1.00, 1.00)
$\exp(\beta_3)$= 2.00	1.00		(0.99, 1.00)	
$\exp(\beta_1)$= 0.40	1.00		(1.00, 1.00)	
$\exp(\beta_2)$= 2.50	1.00	1.00	(1.00, 1.00)	(1.00, 1.00)
$\exp(\beta_3)$= 2.50	1.00		(1.00, 1.00)	
$\exp(\beta_1)$= 0.33	1.00		(1.00, 1.00)	
$\exp(\beta_2)$= 3.00	1.00	1.00	(1.00, 1.00)	(1.00, 1.00)
$\exp(\beta_3)$= 3.00	1.00		(1.00, 1.00)	

Strong Correlation				
exp(β1)= 1.00	0.09		(0.04, 0.11)	
exp(β2)= 1.00	0.07	0.07	(0.05, 0.05)	(0.23, 0.05)
exp(β3)= 1.00	0.06		(0.05, 0.18)	
exp(β1)= 0.67	0.95		(0.73, 0.89)	
exp(β2)= 1.50	0.98	1.00	(0.83, 0.80)	(0.80, 0.93)
exp(β3)= 1.50	0.95		(0.72, 0.89)	
exp(β1)= 0.50	1.00		(0.85, 0.99)	
exp(β2)= 2.00	1.00	1.00	(0.96, 1.00)	(0.93, 1.00)
exp(β3)= 2.00	1.00		(0.86, 1.00)	
exp(β1)= 0.40	1.00		(0.91, 1.00)	
exp(β2)= 2.50	1.00	1.00	(0.99, 1.00)	(1.00, 1.00)
exp(β3)= 2.50	1.00		(0.92, 1.00)	
exp(β1)= 0.33	1.00		(0.93, 1.00)	
exp(β2)= 3.00	1.00	1.00	(1.00, 1.00)	(1.00, 1.00)
exp(β3)= 3.00	1.00		(0.99, 1.00)	

Figure S1. Estimated Chemical Weights for Non-Significant Chemical Groups.

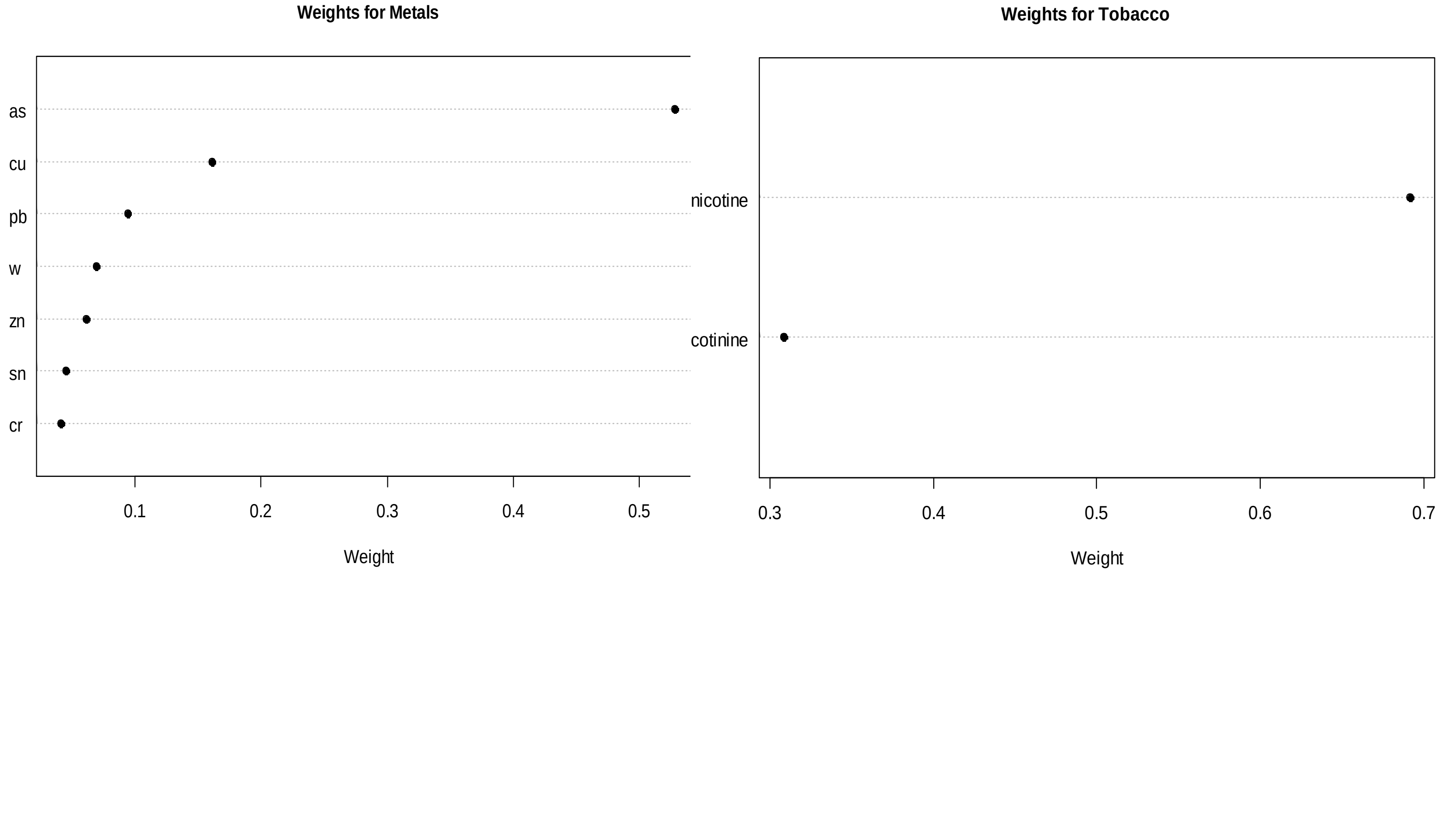

Supplement: Supplementary file 1 [file ijerph-18-00504-s001.pdf]
